# Construction of Recombinant *Lactiplantibacillus plantarum* NC8 Expressing ETEC FaeC and LngC and Preliminary Evaluation of Immune Responses in an ETEC-Challenged Piglet Model

**DOI:** 10.3390/vetsci13101098

**Published:** 2026-10-15

**Authors:** Tianming Niu, Tianyuan Niu, Hongyan Wang, Mingxiao Liu, Kuipeng Gao, Yupeng Wu, Chenxi Jiang, Shi Zhang, Qiong Wu, Shuzhen Zhang, Wentao Yang, Chunwei Shi, Zhipeng Li, Chunfeng Wang

**Affiliations:** 1 College of Veterinary Medicine, Jilin Agricultural University, Changchun 130118, China; 2 Jilin Provincial Engineering Research Center of Animal Probiotics, Jilin Provincial Key Laboratory of Animal Microecology and Healthy Breeding, Jilin Agricultural University, Changchun 130118, China; 3 Key Laboratory of Animal Production and Product Quality Safety of the Ministry of Education, Jilin Agricultural University, Changchun 130118, China; 4 Agriculture and Animal Husbandry Development Center of Tongliao City, Tongliao 028000, China; 5 College of Animal Science and Technology, Jilin Provincial Engineering Research Center of Animal Probiotics, Jilin Agricultural University, 2888 Xincheng Street, Changchun 130118, China

**Keywords:** *Enterotoxigenic Escherichia coli*, *Lactiplantibacillus plantarum NC8*, FaeC, LngC, mucosal immunity, oral vaccine, piglet diarrhea

## Abstract

*Enterotoxigenic Escherichia coli* (ETEC) is a major pathogen responsible for diarrhea and mortality in piglets, causing substantial economic losses to the swine industry. Its pathogenicity relies on the synergistic action of colonization factors and enterotoxins, which mediate intestinal colonization and induce secretory diarrhea. Commercial vaccines against ETEC remain limited, and their protective efficacy in piglets is often unsatisfactory. Consequently, probiotics have attracted increasing attention as mucosal immunomodulatory agents for the prevention and control of ETEC infection. In this study, ETEC, the minor subunit located at the tip of the fimbriae, FaeC, and the fimbrial assembly accessory protein, LngC, were individually inserted into *Lactiplantibacillus plantarum NC8* using the anchoring expression vector pPG612.1, successfully generating the recombinant strains NC8/pPG612.1-FaeC and NC8/pPG612.1-LngC. Western blot analysis confirmed that both recombinant strains correctly expressed the target proteins upon lactose induction, and their growth curves exhibited no significant differences from those of the wild-type strain, indicating that genetic modification did not compromise bacterial proliferation. To evaluate the immune efficacy of the recombinant strains, thirty-six piglets were randomly assigned to six groups: PBS control, ETEC challenge, empty vector, LngC immunization, FaeC immunization, and combined immunization (MIX). After seven oral gavage immunizations, piglets were challenged with ETEC and euthanized on day 7 post-challenge for sample collection. The results showed that immunized piglets resumed body weight gain by day 4 after challenge, whereas the ETEC group exhibited sustained weight loss. Histopathological examination revealed relatively intact jejunal villus architecture and markedly less severe mucosal damage in immunized groups compared with the ETEC-challenged group. RT-qPCR analysis of intestinal tissues and ELISA analysis of serum showed that immunization with the recombinant strains significantly decreased the mRNA expression of pro-inflammatory cytokines (TNF-α, IL-6, and IL-8) in intestinal tissues and their protein levels in serum, while increasing both the mRNA expression and protein levels of the anti-inflammatory cytokine IL-10. Flow cytometric analysis revealed that immunization with the recombinant strains significantly increased the frequencies of CD4^+^IFN-γ^+^ and CD4^+^IL-4^+^T cells and CD21^+^ B cells in MLNs, LPLs, and spleen compared with the control groups. The increased CD4^+^IFN-γ^+^ T-cell population indicated enhanced Th1-mediated cellular immunity, whereas the increased CD4^+^IL-4^+^ T-cell population suggested activation of Th2-associated humoral immunity. The expansion of CD21^+^B cells further indicated enhanced B-cell activation. Collectively, these findings suggest that the recombinant strains induced coordinated mucosal and systemic immune responses. Additionally, the secretory immunoglobulin A (sIgA) concentration in the jejunal contents was markedly increased, suggesting that the intestinal mucosal immune system was effectively activated. Collectively, these findings indicate that NC8/pPG612.1-FaeC and NC8/pPG612.1-LngC can elicit robust mucosal immune responses and alleviate diarrhea induced by enterotoxigenic *Escherichia coli* (ETEC) in piglets, providing a scientific basis for the development of oral probiotic-based vaccines against ETEC infection in swine.

## Simple Summary

To address ETEC-induced diarrhea and mortality in piglets and the limited protection afforded by commercial vaccines, this study constructed recombinant Lactiplantibacillus plantarum NC8 strains expressing ETEC antigens FaeC and LngC and evaluated their immunoprotective effects. The recombinant strains correctly expressed the target proteins upon lactose induction and exhibited normal growth. After immunization and ETEC challenge, piglets in the recombinant groups showed faster body weight recovery, milder jejunal damage, decreased pro-inflammatory cytokines, increased IL-10 and sIgA levels, and enhanced T/B cell activation in MLNs, LPLs, and spleen. These findings indicate that both recombinant strains induce mucosal and systemic immunity and alleviate ETEC-induced diarrhea, providing a basis for developing oral probiotic vaccines to reduce economic losses in the swine industry.

## 1. Introduction

*Enterotoxigenic Escherichia coli* (ETEC) is one of the most important bacterial pathogens causing diarrhea in humans and animals. *E. coli* is a genetically diverse species that includes commensal, environmental, zoonotic, and pathogenic strains; among the pathogenic groups, ETEC is a major cause of acute watery diarrhea. In developing regions, diarrhea remains a leading cause of childhood mortality [1]. Populations in endemic areas are at increased risk because of frequent consumption of food or drinking water contaminated with ETEC. In the swine industry, ETEC strains expressing F4 (K88) fimbriae are common causative agents of neonatal diarrhea and post-weaning diarrhea (PWD), particularly in intensive production systems. Epidemiological surveys have shown that ETEC F4 strains account for approximately 26.4% of piglet diarrhea cases [2]. The severity of ETEC infection in piglets is closely related to age, with 1- to 3-day-old piglets being the most susceptible [3,4]. The infection is highly contagious, and a single infected piglet can rapidly transmit the pathogen to littermates. In addition, piglets born to primiparous sows often have the highest infection rates.

Clinically, ETEC infection in piglets primarily presents as yellow or white watery diarrhea, which is characterized by profuse diarrhea caused by enterotoxin-induced disruption of the intestinal barrier, leading to net fluid secretion into the intestinal lumen. Mechanistically, ETEC colonizes the small intestine via fimbrial adhesins, including F4, and secretes enterotoxins such as heat-labile toxin (LT) and heat-stable toxins (STa/STb). These toxins alter ion transport in enterocytes, promoting chloride secretion and inhibiting sodium absorption, ultimately leading to dehydration, metabolic acidosis, and growth retardation [5]. These pathophysiological changes can result in increased mortality and substantial economic losses in the swine industry.

Given the high prevalence of ETEC and the increasing concern regarding antibiotic resistance, there is an urgent need for safe and effective prevention strategies. Mucosal vaccines and probiotic-based delivery systems have attracted considerable attention because they can stimulate intestinal mucosal immunity and reduce ETEC-induced diarrhea [6,7].

The mucosal immune system, enriched with lymphoid and non-lymphoid cells, serves as the first line of defense against gastrointestinal pathogens. Upon pathogen invasion or vaccination, these immune cells orchestrate both innate and adaptive immune responses. Growing evidence has highlighted the critical role of mucosal immunity in vaccine development [8]. Lactic acid bacteria (LAB), particularly *Lactiplantibacillus* species, have been extensively studied for their immunotherapeutic properties, which are closely linked to their immunogenicity and immunomodulatory capacity [9,10]. To date, numerous LAB-based vaccine candidates have been developed [10,11,12,13]. *Lactiplantibacillus plantarum NC8,* in particular, has emerged as a well-established antigen delivery vehicle due to its favorable attributes, including a high safety profile (generally recognized as safe, GRAS), lack of endotoxin, ease of genetic manipulation, competitive exclusion of enteropathogens, strong adhesion to intestinal epithelium, a thin cell wall facilitating surface display, and absence of native plasmids that might interfere with recombinant constructs [14,15,16,17].

Given the pathogenic mechanisms of ETEC, the unique features of intestinal mucosal immunity, and the advantages of *Lactiplantibacillus plantarum* (formerly *Lactobacillus plantarum*) NC8 as an antigen delivery vector, this study aimed to construct novel recombinant probiotic strains expressing protective ETEC antigens. Specifically, we selected FaeC, the minor subunit located at the tip of the fimbriae, and the fimbrial assembly accessory protein, LngC, as protective antigen genes. FaeC is the minor subunit located at the tip of the fimbriae of F4 that mediates bacterial adhesion to porcine intestinal epithelial cells, whereas LngC is an immunogenic outer membrane protein associated with fimbriae. These two antigens are therefore promising targets for inducing specific mucosal immune responses against ETEC. The two genes were inserted into the surface-anchoring vector pPG612.1, which allows efficient display of heterologous antigens on the bacterial surface, and the resulting constructs were transformed into *Lactiplantibacillus plantarum* NC8 to generate the recombinant strains NC8/pPG612.1-FaeC and NC8/pPG612.1-LngC. The immune effects of these recombinant strains were systematically evaluated through immunization and subsequent ETEC challenge experiments in piglets. Collectively, this study provides a scientific basis for the development of oral probiotic-based vaccines against ETEC-induced diarrhea in swine.

## 2. Materials and Methods

### 2.1. Bacterial Strains, Plasmids, and Culture Conditions

The bacterial strains employed in this study included *Escherichia coli MC1061* and *Lactiplantibacillus plantarum NC8*. *E. coli MC1061* was used as the host strain for plasmid propagation and maintenance, while *Lactiplantibacillus plantarum NC8*, preserved at the College of Veterinary Medicine, Jilin Agricultural University, served as the antigen delivery vehicle. The expression vector pPG612.1 and the recombinant plasmids pUC-FaeC and pUC-LngC, which harbor the ETEC genes faeC and lngC, respectively, were custom-synthesized by Sangon Biotech (Shanghai) Co., Ltd., Shanghai, China. *E. coli MC1061* was routinely cultivated aerobically in Luria–Bertani (LB) broth at 37 °C with continuous shaking (200 rpm). *Lactiplantibacillus plantarum NC8* was propagated statically in de Man, Rogosa, and Sharpe (MRS) medium at 37 °C under anaerobic conditions. For plasmid selection, chloramphenicol (10 μg/mL) was supplemented for pPG-612-derived constructs, and ampicillin (100 μg/mL) was used for pUC-derived plasmids, as appropriate. The challenge strain used in this study was an F4-positive enterotoxigenic *Escherichia coli* (ETEC) standard strain (CVCC196), which was maintained at the College of Veterinary Medicine, Jilin Agricultural University, Changchun, China. This strain was used for challenge infection to preliminarily evaluate the protective effects mediated by FaeC and LngC in piglets immunized with the recombinant strains.

### 2.2. Construction of Recombinant Lactiplantibacillus plantarum Strains

Based on the ETEC FaeC gene sequence (GenBank accession No. AF450247.2) and LngC gene sequence (GenBank accession No. LR883052), specific primers were designed using Primer Premier 5.0 software and synthesized by Sangon Biotech (Shanghai) Co., Ltd. (primer sequences are listed in Table 1). To construct the recombinant plasmids, the pPG612.1 vector, pUC-FaeC, and pUC-LngC plasmids were subjected to double restriction enzyme digestion using AscI and NcoI. The digestion reaction mixture (total volume 50 µL) consisted of 25 µL plasmid DNA, 2 µL AscI, 2 µL NcoI, 5 µL 10× buffer, and 16 µL ddH_2_O, and was incubated at 37 °C for 6 h. The digested products were resolved by 1% agarose gel electrophoresis at 110 V for 20 min, and the linearized vector and target gene fragments were purified using a gel extraction kit. The purified linearized vector and target inserts were ligated using T4 DNA ligase in a reaction mixture (total volume 10 µL) containing 2 µL linearized vector, 5 µL insert fragment, 1 µL 10× T4 DNA Ligase Buffer, 1 µL T4 DNA Ligase, and 1 µL ddH_2_O, followed by overnight incubation at 16 °C. The ligation products were subsequently transformed into *E. coli MC1061* competent cells via the heat-shock method. Positive transformants were selected on LB agar plates containing appropriate antibiotics. Single colonies were picked and inoculated into 5 mL LB liquid medium and incubated at 37 °C with shaking at 200 rpm. Recombinant plasmids were extracted using a plasmid miniprep kit. The presence of the FaeC and LngC genes in the recombinant plasmids was confirmed by PCR using the specific primers listed in Table 1. The PCR reaction mixture (total volume 50 µL) comprised 1 µL plasmid template, 25 µL 2× PrimeSTAR^®^ Mix, 1 µL each of forward and reverse primers, and 22 µL ddH_2_O. The amplification protocol was as follows: initial denaturation at 94 °C for 5 min; 35 cycles of denaturation at 94 °C for 40 s, annealing at 60 °C for 40 s, and extension at 72 °C for 1 min; followed by a final extension at 72 °C for 10 min. Subsequently, the recombinant plasmids pPG612.1-FaeC and pPG612.1-LngC were further verified by double restriction enzyme digestion with AscI and NcoI. Finally, the verified recombinant plasmids pPG612.1-FaeC and pPG612.1-LngC were introduced into *Lactiplantibacillus plantarum NC8* competent cells by electroporation. Positive transformants were screened on MRS agar plates containing chloramphenicol (10 µg/mL), and the integration of the target genes was confirmed by PCR using the aforementioned primers.

### 2.3. Verification of Target Protein Expression in Recombinant Strains

To evaluate the growth characteristics, the successfully identified recombinant strains were activated by inoculating 100 μL of the glycerol stock into 5 mL of MRS broth and incubated anaerobically overnight at 37 °C. Subsequently, the overnight cultures were subcultured at a 1% (*v*/*v*) inoculum into 100 mL of fresh MRS broth and incubated at 37 °C with shaking at 200 rpm. Aliquots (2 mL) were collected at 2 h intervals throughout a 24 h cultivation period to measure the optical density at 600 nm (OD_600_). The growth curves were plotted using GraphPad Prism software. For protein expression analysis, recombinant strains were first activated as described above. The activated cultures were then inoculated at 1% (*v*/*v*) into 100 mL of MRS broth supplemented with 2% (*w*/*v*) lactose as an inducer and incubated anaerobically at 37 °C overnight. Bacterial cells were harvested by centrifugation (8000× *g*, 4 °C, 10 min), washed twice with sterile phosphate-buffered saline (PBS), and resuspended in an appropriate volume of PBS. The total protein concentration of the bacterial suspensions was determined using the BCA protein assay kit. Subsequently, 400 μL of the resuspended bacterial solution was mixed with 100 μL of 5× SDS-PAGE loading buffer, boiled for 10 min to denature the proteins, and then centrifuged at 12,000 rpm for 1 min. The supernatants were collected and stored at −40 °C until further use. Finally, the expression of the target proteins (FaeC and LngC) in the recombinant *Lactiplantibacillus plantarum NC8* strains was verified by Western blotting. Equal amounts of total protein were separated by SDS-polyacrylamide gel electrophoresis (SDS-PAGE) and electrotransferred onto polyvinylidene difluoride (PVDF) membranes. The membranes were blocked with 5% skim milk and subsequently incubated with a specific HRP-conjugated anti-6 × His tag monoclonal antibody (Invitrogen, cat. no. MA1-21315-HRP; Thermo Fisher Scientific, Waltham, MA, USA) against the target proteins. Since the antibody was HRP-conjugated, no secondary antibody was required. Immunoreactive bands were visualized using an enhanced chemiluminescence (ECL) substrate and imaged with a chemiluminescence detection system.

### 2.4. Animal Grouping, Immunization, and Challenge Protocol

In this experiment, a total of 36 piglets born to three Large White (Yorkshire) sows were used. The piglets were randomly assigned to six experimental groups, with male and female piglets balanced among the groups. Each piglet was individually housed in a separate temperature-controlled chamber and fed a commercial pig milk replacer (Zhongmei Puke; [Zhongmei Puke limited company, Zhengzhou, Henan China]) under identical feeding and management conditions. All animal procedures were approved by the Animal Care and Use Committee of [the Animal Welfare and Ethics Committee of Jilin Agricultural University, Changchun, Jilin Province, China] (approval no. [2024 12 23 001]). Grouped as follows: (i) PBS control group (PBS), orally administered 1 mL of sterile PBS; (ii) ETEC challenge group (ETEC), orally administered 1 mL of ETEC suspension at 5.34 × 10^7^ CFU/mL; (iii) empty vector group (pPG), orally administered 1 mL of NC8/pPG612.1 at 1 × 10^9^ CFU/mL; (iv) FaeC immunization group (FaeC), orally administered 1 mL of NC8/pPG612.1-FaeC at 1 × 10^9^ CFU/mL; (v) LngC immunization group (LngC), orally administered 1 mL of NC8/pPG612.1-LngC at 1 × 10^9^ CFU/mL; and (vi) combined immunization group (MIX), orally administered 1 mL of a 1:1 mixture of NC8/pPG612.1-FaeC and NC8/pPG612.1-LngC (each at a final concentration of 0.5 × 10^9^ CFU/mL, total 1 × 10^9^ CFU/mL). For immunization, bacterial cultures were harvested at the logarithmic growth phase, washed twice with sterile PBS, and resuspended in PBS to a final concentration of 1 × 10^9^ CFU/mL, determined by colony-counting assays using the relationship between OD_600_ and viable cell counts. Each piglet received 1 mL of the corresponding bacterial suspension via oral gavage every other day (with a 1-day interval between immunizations) for a total of seven immunizations. One day after the final immunization, all piglets except those in the PBS group were orally challenged with ETEC at a dose of 1 mL per kilogram of body weight of a suspension containing 5.34 × 10^7^ CFU/mL. The PBS group received an equivalent volume of sterile PBS. Following the challenge, all piglets were monitored daily for 7 days, and body weights were recorded to calculate the body weight gain rate. At 7 days post-challenge, piglets were weighed and blood samples were collected via jugular vein puncture. Subsequently, all animals were humanely euthanized, and intestinal tissues (jejunum, ileum), mesenteric lymph nodes, spleen, and jejunal contents were aseptically collected for further analyses.

After the ETEC challenge, the fecal appearance of piglets in each group was observed daily at a fixed time by [two trained observers blinded to group allocation]. Fecal consistency was scored using a 5-point scale ranging from −2 to 2 as follows: a score of 2 was assigned to cylindrical or pellet-shaped feces that were well-formed and of appropriate hardness; a score of 1 to soft, loose feces that remained formed with normal moisture; a score of 0 to soft, loose feces that remained formed with abnormal moisture; a score of −1 to thick, semi-liquid, unformed feces without obvious separation of solid and liquid; and a score of −2 to watery feces with separation of solid and liquid and a runny consistency. In this study, diarrhea was defined as a fecal score ≤ 0. The daily fecal scores were recorded for each piglet, and the average fecal score was calculated.

Fecal samples were collected daily from piglets in each group after the ETEC challenge. Approximately 1 g of feces was weighed and homogenized in 9 mL of sterile buffered peptone water (BPW) by vortexing to obtain a 10^−1^ homogenate. The homogenate was then serially diluted 10-fold in sterile BPW to obtain dilutions of 10^−2^ to 10^−7^, and the dilution range was adjusted according to the expected bacterial concentration. An aliquot of 100 μL of the appropriate dilution was spread onto *E. coli* chromogenic medium plates. The plates were incubated aerobically at 37 °C for 18–24 h. After incubation, plates containing 30–300 colonies were selected for counting. The number of ETEC colonies was recorded, and the results were expressed as colony-forming units (CFU) per gram of feces (CFU/g) using the following formula:

CFU/g feces = (number of colonies × dilution factor × 100)/1 g

### 2.5. Histopathological Examination

For histopathological evaluation, jejunal tissue samples were collected immediately after euthanasia, rinsed with sterile PBS to remove intestinal contents, and fixed in 4% paraformaldehyde solution for at least 48 h (optimal fixation time: 3–5 days) at room temperature. Following fixation, tissue specimens were trimmed to obtain uniform cross-sections of the intestinal wall using a scalpel. The fixed tissues were dehydrated through a graded ethanol series as follows: 70% ethanol for 3.5 h, 80% ethanol for 3.5 h, 85% ethanol for 3.5 h, 90% ethanol for 3.5 h, 95% ethanol I overnight, 95% ethanol II for 2 h, 100% ethanol I for 2 h, and 100% ethanol II for 3 h. After dehydration, the specimens were cleared in two changes of xylene (xylene I and xylene II) for 5 min each and subsequently infiltrated with paraffin at 58 °C through three changes of molten paraffin (wax I, II, and III) for 30 min each. The infiltrated tissues were then embedded in paraffin blocks using an embedding center. Serial sections were cut at a thickness of 3 μm using a rotary microtome and floated in a 42 °C water bath to ensure complete flattening, then mounted onto glass slides. The sections were dried in an oven at 80 °C for 1 h. For hematoxylin and eosin (H&E) staining, the deparaffinized and rehydrated sections were processed as follows: xylene I for 8 min, xylene II for 8 min, 100% ethanol I for 1 min, 100% ethanol II for 1 min, 95% ethanol for 1 min, 80% ethanol for 1 min, and 70% ethanol for 1 min. Subsequently, sections were stained with hematoxylin for 5 min, differentiated in 0.5% acid-alcohol for 5 s, rinsed with 0.5% ammonia water (alkaline water) for 2 min, and counterstained with 0.5% eosin aqueous solution for 5 min. The stained sections were then dehydrated through a graded ethanol series (95% ethanol for 1 min, 100% ethanol I for 1 min, and 100% ethanol II for 1 min), cleared in xylene I for 2 min and xylene II for 2 min, and finally mounted with neutral resin. All stained slides were observed and photographed under a Nikon ECLIPSE Ci-L light microscope (Nikon Corporation, Tokyo, Japan) to evaluate the morphological integrity of jejunal villi and the degree of pathological damage.

Images were captured under a light microscope at 200× magnification and analyzed with ImageJ 4.2 software. Measurements were performed blindly with respect to group allocation, and fields of view were randomly selected to avoid bias. Well-oriented, longitudinally sectioned villus–crypt units without oblique sectioning were measured. Villus height was measured from the villus tip to the villus–crypt junction, and crypt depth from the villus–crypt junction to the crypt base. Ten intact villus–crypt units were randomly measured per section, and the V/C ratio was calculated. Data are expressed as mean ± SD.

### 2.6. Enzyme-Linked Immunosorbent Assay (ELISA)

#### 2.6.1. Quantification of sIgA in Jejunal Contents

After euthanasia, the abdominal cavity was opened via a midline laparotomy under aseptic conditions. The jejunum was exposed, and both ends of the selected jejunal segment were immediately ligated with sterile surgical sutures. The ligated segment was then excised and placed in a sterile petri dish for subsequent analysis. The intestinal contents were gently expressed into pre-weighed 5 mL centrifuge tubes, and the sample weight was recorded. Three volumes (*w*/*v*) of ice-cold phosphate-buffered saline (PBS, pH 7.4) supplemented with a protease inhibitor cocktail were added to each sample, followed by thorough vortexing to homogenize the contents. The mixtures were then centrifuged at 4000 rpm for 15 min at 4 °C. The supernatants were collected, aliquoted, and stored at −80 °C until further analysis. The secretory immunoglobulin A (sIgA) levels in the jejunal content supernatants were measured using a commercially available porcine-specific sIgA ELISA kit (cat. no. MM-36234O1, Shanghai Enzyme-linked Biotechnology Co., Ltd., Shanghai, China) according to the manufacturer’s instructions. Briefly, serial dilutions of standards and appropriately diluted samples were added to 96-well plates pre-coated with anti-pig sIgA monoclonal antibody and incubated at 37 °C. After washing, biotinylated detection antibody and HRP-conjugated streptavidin were sequentially added, followed by TMB substrate for color development. The reaction was terminated with 2 M H_2_SO_4_, and the absorbance was read at 450 nm using a microplate reader. The sIgA concentration in each sample was calculated from the standard curve and expressed as micrograms per gram of intestinal content (μg/g).

#### 2.6.2. Detection of Antigen-Specific IgG Antibodies

The B-cell epitopes of ETEC LngC and FaeC were predicted using BepiPred-2.0 and BepiPred-3.0. Candidate epitopes were screened according to the following criteria: a BepiPred score > 0.5, predicted surface accessibility, high hydrophilicity, and absence of transmembrane regions. Linear peptides of 10–15 amino acids (aa) were selected as candidate B-cell epitope peptides. The selected epitope peptides were RENMEYTDGT for FaeC and AYQRDGKYPDFV for LngC. These peptides were commercially synthesized by Sangon Biotech (Shanghai) Co., Ltd. (Shanghai, China) with a purity of 99.99% as determined by high-performance liquid chromatography (HPLC).

On day 14 after immunization, serum samples were collected from piglets in each group. Blood samples were incubated at 37 °C for 2 h and then centrifuged at 3500 rpm for 10 min at 4 °C. Serum was collected, and the levels of specific IgG antibodies against the ETEC antigens FaeC and LngC were determined by ELISA. The ELISA procedure was as follows. Serum samples from each group were serially diluted twofold from 1:2 to 1:512. The synthesized B-cell epitope peptides of the ETEC antigens FaeC and LngC were diluted to 0.125 µg/mL and added to 96-well ELISA plates at 200 µL per well, followed by incubation at 4 °C for 12 h. After incubation, the liquid was discarded, and the plates were washed three times with 300 µL of PBS containing 0.5% Tween 20 (PBST) and blotted dry on absorbent paper. The plates were then blocked with 300 µL per well of 0.2% BSA blocking solution and incubated at 37 °C for 2 h. After washing as described above, 300 µL of diluted serum samples were added to each well and incubated at 37 °C for 1.5 h. After washing, the HRP-conjugated anti-pig IgG secondary antibody was diluted 1:5000 and added to the wells, followed by incubation at 37 °C for 1 h. After washing, 100 µL of TMB substrate was added to each well, and the plates were incubated in the dark at room temperature for 10 min. The reaction was terminated with diluted sulfuric acid, and the absorbance was measured at 450 nm using a microplate reader.

#### 2.6.3. Quantification of Cytokine Levels in Serum

At 7 days after the final challenge, piglets were fixed in a supine position, and blood samples were collected from the left jugular vein groove (precava region) using a 5 mL syringe inserted at a 45° angle. The blood was immediately transferred into 5 mL centrifuge tubes and allowed to clot by standing at 37 °C for 1 h. Subsequently, the samples were centrifuged at 4000 rpm for 15 min at 4 °C, and the serum supernatants were carefully collected, aliquoted, and stored at −80 °C until use. The levels of pro-inflammatory cytokines, including tumor necrosis factor-alpha (TNF-α), interleukin-6 (IL-6), and interleukin-1β (IL-1β), as well as the anti-inflammatory cytokine interleukin-10 (IL-10), in serum samples were quantified using porcine-specific ELISA kits (e.g., from a commercial supplier) according to the manufacturers’ protocols. All samples were measured in duplicate, and the cytokine concentrations were calculated from standard curves generated with recombinant porcine cytokine standards. Results are expressed as picograms per milliliter (pg/mL).

### 2.7. Quantification of Cytokine mRNA Expression in Jejunum by Quantitative Real-Time PCR

Total RNA was extracted from jejunal tissue samples stored at −80 °C using the TRIzol^®^ reagent method. Approximately 50 mg of tissue was homogenized in 1 mL of TRIzol using an electric tissue grinder until a homogeneous suspension was obtained. The homogenates were centrifuged at 12,000 rpm for 10 min at 4 °C, and the supernatants were transferred to fresh 1.5 mL RNase-free centrifuge tubes. After incubation at room temperature for 5 min, 0.2 mL of chloroform was added to each tube, followed by vigorous vortexing for 15 s and further incubation at room temperature for 5 min. The samples were then centrifuged at 12,000 rpm for 15 min at 4 °C, resulting in three distinct phases. The upper aqueous phase containing total RNA was carefully transferred to a new RNase-free tube. RNA was precipitated by adding two volumes of isopropanol (2:1, *v*/*v*) to the aqueous phase, mixed by inverting, and incubated on ice for 5 min, followed by centrifugation at 12,000 rpm for 10 min at 4 °C. The supernatant was discarded, and the RNA pellet was washed with 1 mL of pre-chilled 75% ethanol by vortexing and centrifuged at 12,000 rpm for 5 min at 4 °C. After removing the supernatant, the pellet was air-dried in a laminar flow hood and dissolved in 30 μL of DEPC-treated water by incubation in a metal bath at 37 °C for 15 min. The RNA concentration and purity (A_260_/A_280_ ratio) were determined using a spectrophotometer (NanoDrop, Thermo Fisher Scientific, USA), and RNA integrity was confirmed by agarose gel electrophoresis. First-strand cDNA was synthesized using a commercial reverse transcription kit (BioSharpBioSharp Biotechnology Co., Ltd.Hangzhou.China, containing genomic DNA eraser) according to the manufacturer’s instructions. The reverse transcription reaction mixture (total volume 40 μL) comprised 16 μL of 5× RT MasterMix, 4 μL of 20× Oligo dT and Random Primer, 0.1–4 μg of total RNA, and RNase-free water to make up the final volume. The reaction was performed at 37 °C for 15–30 min, followed by 85 °C for 5 min to inactivate the enzyme. The resulting cDNA was stored at −20 °C until further use. Quantitative real-time PCR (qPCR) was performed using a BioSharp SYBR Green qPCR kit on a real-time PCR detection system (Applied Biosystems 7500 Real-Time PCR System, Thermo Fisher Scientific, USA). Porcine GAPDH was used as the internal reference gene. Specific primers targeting porcine TNF-α, IL-6, IL-8, and IL-10 genes were designed based on sequences deposited in the NCBI database (primer sequences are listed in Table 2). Each qPCR reaction (total volume 20 μL) contained 2 μL of cDNA template, 1 μL each of forward and reverse primers (10 μM), 8 μL of 2× Universal SYBR qPCR Mix, 0.4 μL of ROX Reference Dye (25 μM), and RNase-free water to 20 μL. The amplification protocol was as follows: initial denaturation at 95 °C for 5 min, followed by 40 cycles of 95 °C for 10 s, 60 °C for 30 s, and 72 °C for 30 s. All samples were run in triplicate, and the relative expression levels of target genes were calculated using the 2^−^ΔΔCt method after normalization to GAPDH.

### 2.8. Flow Cytometric Analysis

#### 2.8.1. Preparation of Single-Cell Suspensions

Spleen (SP), mesenteric lymph nodes (MLN), and intestinal lamina propria lymphocytes (LPL) were aseptically collected from piglets at necropsy. A 200-mesh stainless steel sieve was placed over a 35 mm cell culture dish containing 1 mL of complete RPMI-1640 medium (supplemented with 10% FBS, 100 U/mL penicillin, and 100 μg/mL streptomycin) and kept at 4 °C. Tissue samples (SP, MLN, and LPL) were placed onto the sieve and gently homogenized using the rubber end of a 1 mL syringe plunger. The homogenates were filtered through the sieve, and the resulting cell suspensions were collected into 1.5 mL centrifuge tubes. For spleen samples, the cell suspension was treated with 1 mL of pre-chilled red blood cell (RBC) lysis buffer to remove erythrocytes, gently mixed, and incubated on ice for 5 min. After adding 500 μL of PBS, the suspension was centrifuged at 500× *g* for 5 min at 4 °C. The supernatant was discarded, and the cell pellet was washed twice with PBS. For MLN and LPL samples, the cell suspensions were directly washed with PBS under the same centrifugation conditions. The final cell pellets from SP, MLN, and LPL samples were resuspended in 1 mL of PBS, and the cell density was determined using a hemocytometer after appropriate dilution. An aliquot of the cell suspension containing the desired cell number was taken, centrifuged at 500× *g* for 5 min at 4 °C, resuspended in 100 μL of PBS, and kept at 4 °C for subsequent antibody staining.

#### 2.8.2. Detection of T-Cell Subsets

To 100 μL of the single-cell suspension, 10 μL of PE-conjugated anti-pig CD4 monoclonal antibody (diluted 1:35) was added, gently mixed, and incubated at 4 °C in the dark for 30 min. Following incubation, 500 μL of PBS was added, and the cells were centrifuged at 500× *g* for 5 min at 4 °C. The supernatant was discarded, and the cell pellet was resuspended in 80 μL of Fixation/Permeabilization solution and incubated at 4 °C in the dark for 45 min. After fixation, 150 μL of Permeabilization Wash buffer was added, and the cells were centrifuged at 500× *g* for 5 min at 4 °C. The supernatant was removed, and 20 μL of a mixture containing APC-conjugated anti-pig IL-4 (diluted 1:35) and PerCP-Cy5.5-conjugated anti-pig IFN-γ (diluted 1:40) antibodies was added, mixed gently, and incubated at 4 °C in the dark for 45 min. Following incubation, 200 μL of Permeabilization Wash buffer was added, and the cells were centrifuged at 500× *g* for 5 min at 4 °C. The supernatant was discarded, and the cell pellet was resuspended in 200 μL of PBS. The suspension was filtered through a nylon mesh into a flow cytometry tube, brought to a final volume of 300 μL with PBS, and stored at 4 °C in the dark until acquisition.

#### 2.8.3. Detection of B Cells

To 100 μL of the single-cell suspension, 10 μL of PE-conjugated anti-pig CD21 monoclonal antibody (diluted 1:40) was added, gently mixed, and incubated at 4 °C in the dark for 45 min. After incubation, 1000 μL of PBS was added, and the cells were centrifuged at 500× *g* for 5 min at 4 °C. The supernatant was discarded, and the cell pellet was resuspended in 200 μL of PBS. The cell suspension was filtered through a nylon mesh into a flow cytometry tube, brought to a final volume of 300 μL with PBS, and stored at 4 °C in the dark until acquisition. All stained samples were analyzed using a flow cytometer. Data were processed using FlowJo 10.0software, and the percentages of positive cells for each marker were determined after excluding debris and doublets based on forward and side scatter profiles.

### 2.9. Statistical Analysis

Statistical analyses were performed using GraphPad Prism 10 software [GraphPad Software, San Diego, CA, USA]. Data are presented as mean ± standard deviation (SD) or standard error of the mean (SEM), as indicated. The individual piglet was considered the experimental unit (biological replicate), and technical replicates were averaged before analysis. Body weight and daily fecal scores were measured repeatedly in the same piglets; therefore, linear mixed-effects models (LMMs) and generalized linear mixed-effects models (GLMMs) were used, respectively. The models included group, time, and group × time interaction as fixed effects. Piglet was included as a random effect to account for repeated measurements, and litter was included as a random effect to account for clustering within litters. For body weight, an LMM with an appropriate covariance structure (e.g., compound symmetry or first-order autoregressive) was fitted. For daily fecal scores, a cumulative link mixed model was used because the scores were ordinal. For diarrhea incidence, a GLMM with a binomial distribution and logit link was used. For fecal ETEC shedding (CFU/g feces), a GLMM with a negative binomial distribution was used. When a significant group × time interaction was detected, post hoc pairwise comparisons were performed using estimated marginal means with Tukey’s adjustment. For single-time-point variables, including villus height, crypt depth, V/C ratio, and serum IgG levels, one-way ANOVA followed by Tukey’s test was used; when litter effects were present, litter was included as a random effect in a mixed model. The 36 piglets were derived from three litters (12 piglets per litter), and piglets from each litter were randomly allocated across the six experimental groups, with two piglets per group per litter, resulting in six piglets per group. All technical replicates were averaged prior to statistical analysis. *p* < 0.05 was considered statistically significant.

## 3. Results

### 3.1. Construction of Recombinant Lactiplantibacillus plantarum NC8 Strains Expressing ETEC Protective Antigens

**Figure 1:**
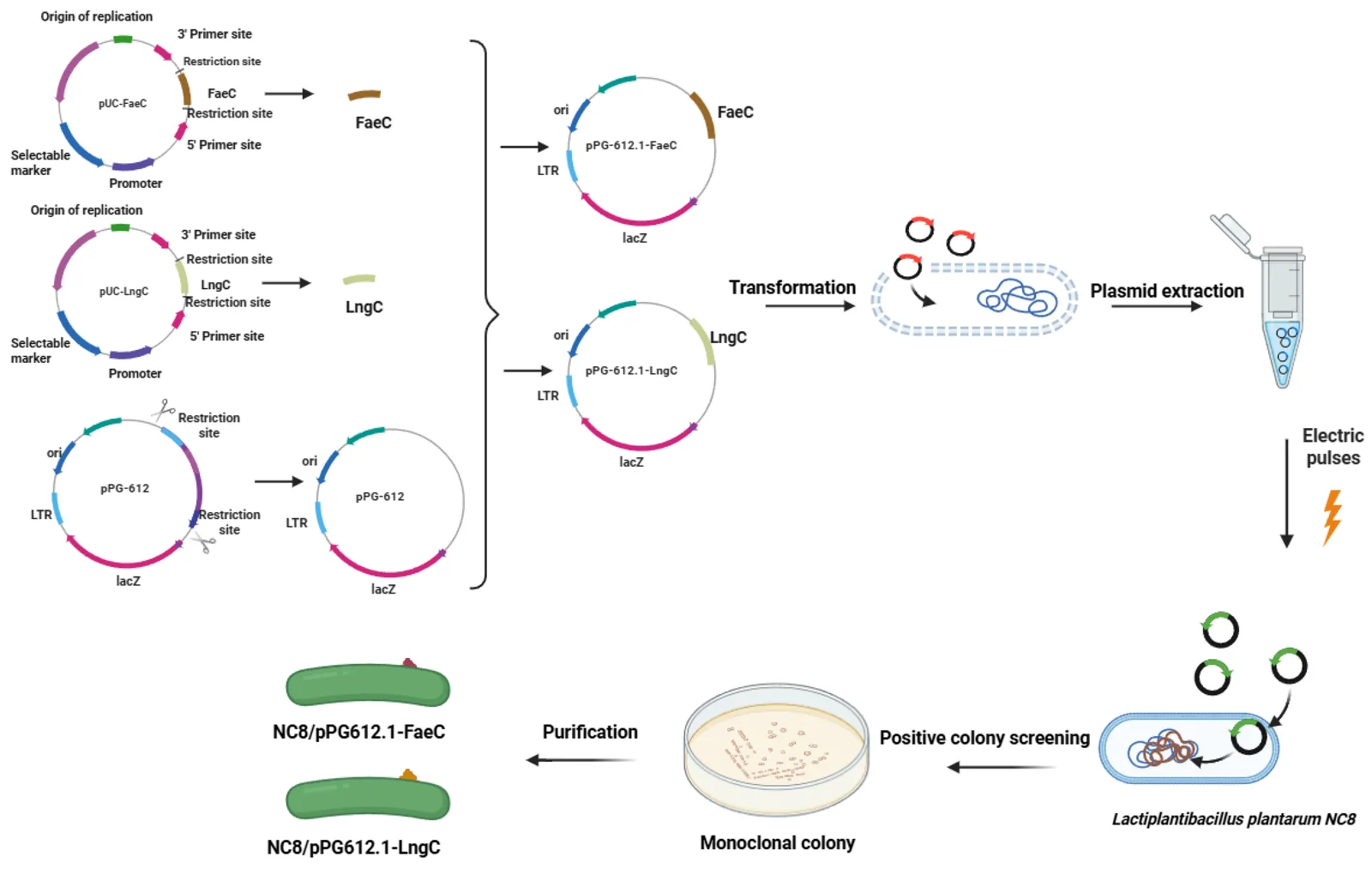
Construction strategy of recombinant Lactobacillus plantarum strains expressing ETEC protective antigens. The FaeC and LngC gene fragments were excised from pUC-FaeC and pUC-LngC by restriction enzyme digestion and separately inserted into the pPG612.1 vector using seamless cloning. The resultant plasmids were propagated in *E. coli MC1061*, and after sequence confirmation, they were transformed into Lactobacillus plantarum NC8, generating the recombinant strains NC8/pPG612.1-FaeC and NC8/pPG612.1-LngC.

**Figure 2:**
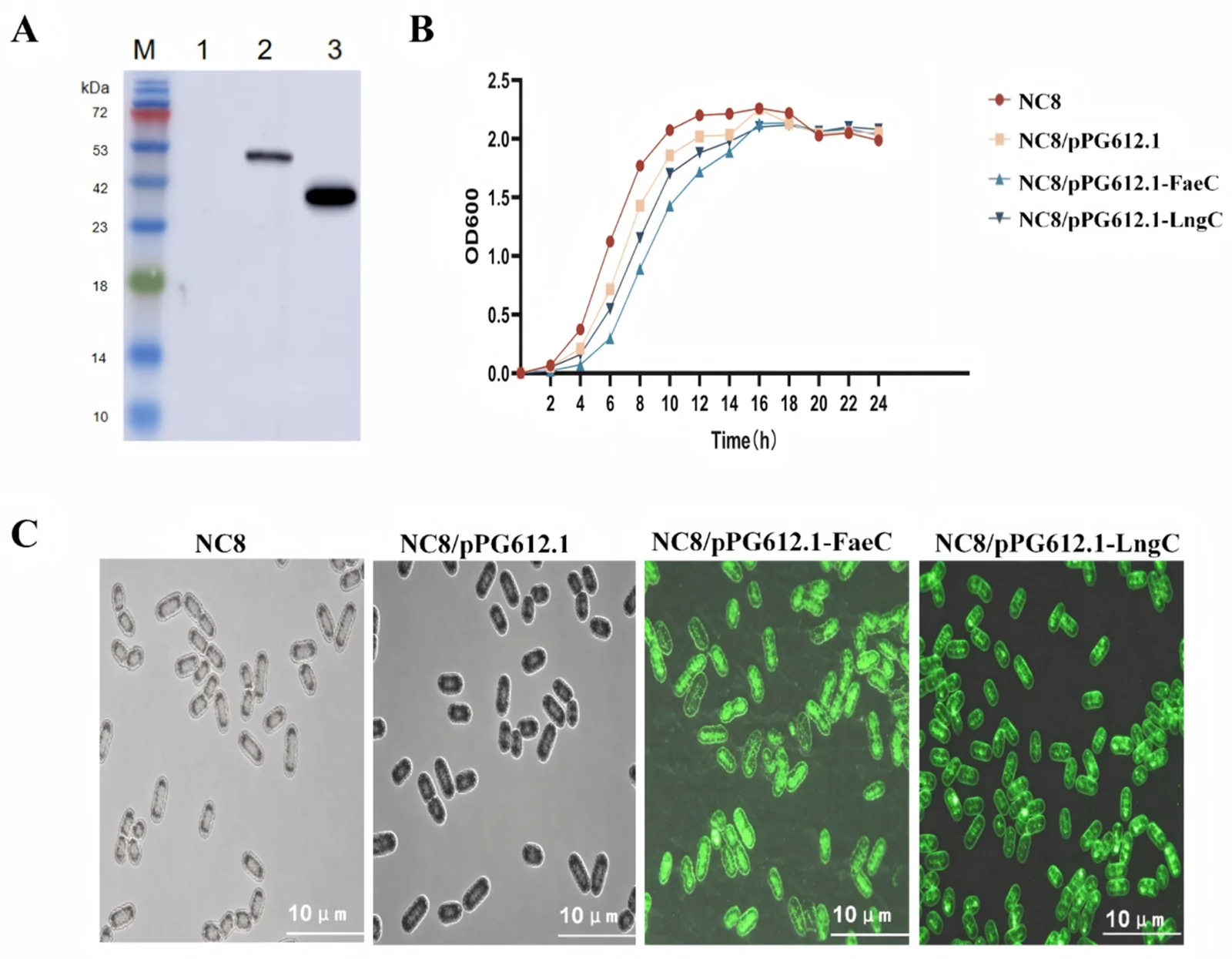
Identification and surface anchoring validation of recombinant proteins expressed in *Lactiplantibacillus plantarum*. (**A**) Western blot analysis detected specific bands of approximately 51 kDa and 40 kDa in NC8/pPG612.1-FaeC and NC8/pPG612.1-LngC, respectively, matching the predicted molecular weights of FaeC and LngC. (**B**) Growth curves of the parental strain NC8, the empty-vector control NC8/pPG612.1, and the two recombinant strains were monitored. (**C**) Immunofluorescence staining with FITC-conjugated anti-Flag monoclonal antibody revealed uniformly distributed strong green fluorescence on the surface of both recombinant strains, confirming successful anchoring of the target proteins to the cell surface.

In this study, the ETEC protective antigen genes FaeC and LngC were amplified and obtained by double restriction enzyme digestion, and subsequently ligated into the cloning/expression vector pPG-612.1. The resulting recombinant plasmids were then introduced into *Lactiplantibacillus plantarum NC8* by electroporation (Figure 1). Two recombinant strains, designated NC8/pPG612.1-FaeC and NC8/pPG612.1-LngC, were successfully constructed, as confirmed by colony PCR (Figure S1). To verify the expression of the target proteins, Western blotting was performed using an anti-Flag mouse monoclonal antibody. Specific immunoreactive bands corresponding to FaeC (approximately 51 kDa) and LngC (approximately 40 kDa) were clearly detected in the respective recombinant strains, whereas no such bands were observed in the control strain harboring the empty vector (Figure 2A). These results demonstrated that both FaeC and LngC were successfully expressed in *Lactiplantibacillus plantarum NC8*. To further visualize the surface localization of the expressed antigens, the recombinant strains were labeled with FITC-conjugated anti-Flag monoclonal antibody and examined under a fluorescence microscope. As shown in Figure 2C, intense green fluorescence was evenly distributed over the entire surface of both recombinant strains, indicating that the target proteins were successfully anchored on the bacterial cell surface. To evaluate whether the heterologous protein expression affected bacterial growth, the growth curves of the recombinant strains were monitored over a 24 h cultivation period. As depicted in Figure 2B, the growth profiles of NC8/pPG612.1-FaeC and NC8/pPG612.1-LngC were comparable to that of the wild-type NC8 strain, suggesting that the expression of the foreign antigens did not impose a significant metabolic burden on the host bacterium.

### 3.2. Immune Efficacy of Recombinant Strains in Piglets

**Figure 3:**
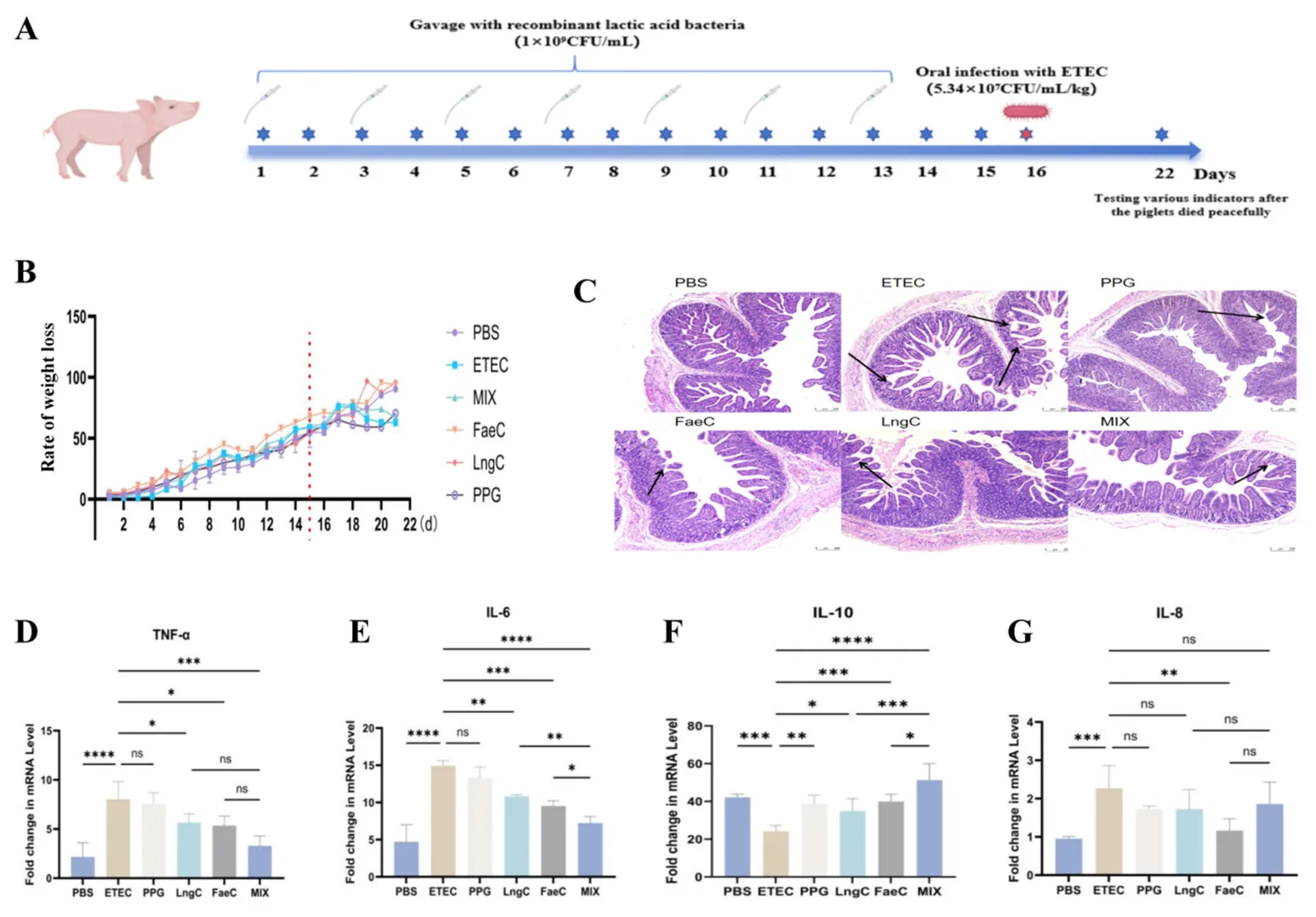
Infection and protection assays in piglets. (**A**) Schematic diagram of the oral gavage and challenge regimen. (**B**) Body weight loss of piglets in each group. (**C**) Histopathological examination of small intestinal sections showing tissue damage. (**D**–**G**) Relative mRNA expression levels of TNF-α (**D**), IL-6 (**E**), IL-10 (**F**), and IL-8 (**G**) in the jejunum of piglets from each group, determined by RT-qPCR. Data are presented as mean ± SEM (n = 6 piglets per group). Statistical significance was assessed by one-way ANOVA compared with the control group; * *p* < 0.05, ** *p* < 0.01, *** *p* < 0.001, **** *p* < 0.0001; NS, not significant.

To evaluate the immunological effects of the recombinant strains NC8/pPG612.1-FaeC and NC8/pPG612.1-LngC, neonatal piglets (1-day-old, *n* = 6 per group) were orally administered with either NC8/pPG612.1-FaeC, NC8/pPG612.1-LngC, or the empty vector control (1 × 10^9^ CFU/mL) every other day for a total of seven immunizations. One day after the final immunization, all piglets except the PBS control group were orally challenged with ETEC at a dose of 1 mL/kg body weight (5.34 × 10^7^CFU/mL) (Figure 3A). On day 7 post-challenge, piglets were weighed, blood samples were collected, and all animals were euthanized to collect Peyer’s patches, spleen, mesenteric lymph nodes, and intestinal tissues. Body weight was recorded daily from the first day of immunization until euthanasia on day 7 post-challenge. As shown in the growth curve (Figure 3B), body weight gain in the ETEC-challenged group decreased continuously from day 4 post-challenge. In contrast, piglets immunized with LngC, FaeC, or the combined MIX regimen exhibited better growth performance than those in the ETEC group and resumed body weight gain from day 4 post-challenge, comparable to the PBS control group. Histopathological examination of the jejunum revealed varying degrees of villus injury in all ETEC-challenged groups (Figure 3C). To evaluate the protective effect of recombinant lactic acid bacteria on intestinal morphology, villus height, crypt depth, and the villus height-to-crypt depth ratio (V/C) were quantified in histological sections of the piglet small intestine. The results demonstrated that the lactic acid bacteria treatment preserved villus integrity and attenuated intestinal villus injury compared with the control group (Supplementary Figure S2). After ETEC challenge, piglets in the ETEC-infected group exhibited evident diarrhea and persistently shed ETEC in their feces. In contrast, piglets in the probiotic and Mix groups showed fecal characteristics similar to those of the PBS control group, with no obvious diarrhea, and their fecal ETEC shedding was markedly reduced during the days after infection. These results are presented in Supplementary Figure S3. The ETEC group displayed the most severe injury, characterized by sparse, fragmented villi with marked structural alterations, in contrast to the intact villus architecture observed in the PBS group. Among the immunized groups, the MIX group exhibited the mildest intestinal damage, with relatively intact villus structures, followed by the LngC and FaeC groups, indicating that the combined immunization provided superior protection. The mRNA expression levels of pro-inflammatory cytokines (TNF-α, IL-6, IL-8) and the anti-inflammatory cytokine IL-10 in jejunal tissues were determined by RT-qPCR (Figure 3D–G). Compared with the ETEC group, the MIX group showed a significant downregulation of TNF-α expression (*p* < 0.001), while the LngC and FaeC groups also exhibited reduced TNF-α levels (*p* < 0.05) (Figure 3D). For IL-6, the MIX group demonstrated a highly significant decrease (*p* < 0.0001), and the LngC and FaeC groups showed significant reductions (*p* < 0.001) compared with the ETEC group. Moreover, IL-6 levels in the MIX group were significantly lower than those in the LngC and FaeC groups (*p* < 0.01) (Figure 3E). Regarding the anti-inflammatory cytokine IL-10, the MIX group exhibited a marked upregulation (*p* < 0.0001), and both the LngC and FaeC groups showed increased IL-10 expression (*p* < 0.001) relative to the ETEC group. Notably, IL-10 levels in the MIX group were significantly higher than those in the LngC and FaeC groups (*p* < 0.001) (Figure 3F). For IL-8, a significant reduction was observed in the FaeC group compared with the ETEC group (*p* < 0.01) (Figure 3G). Collectively, these results indicate that ETEC infection induced a substantial release of pro-inflammatory cytokines in the intestine. Prophylactic immunization with the recombinant strains effectively reduced the secretion of pro-inflammatory cytokines while enhancing anti-inflammatory IL-10 levels, thereby alleviating intestinal inflammation in piglets.

### 3.3. Recombinant Strains Enhance Secretory IgA and IgG Expression in Piglets

**Figure 4:**
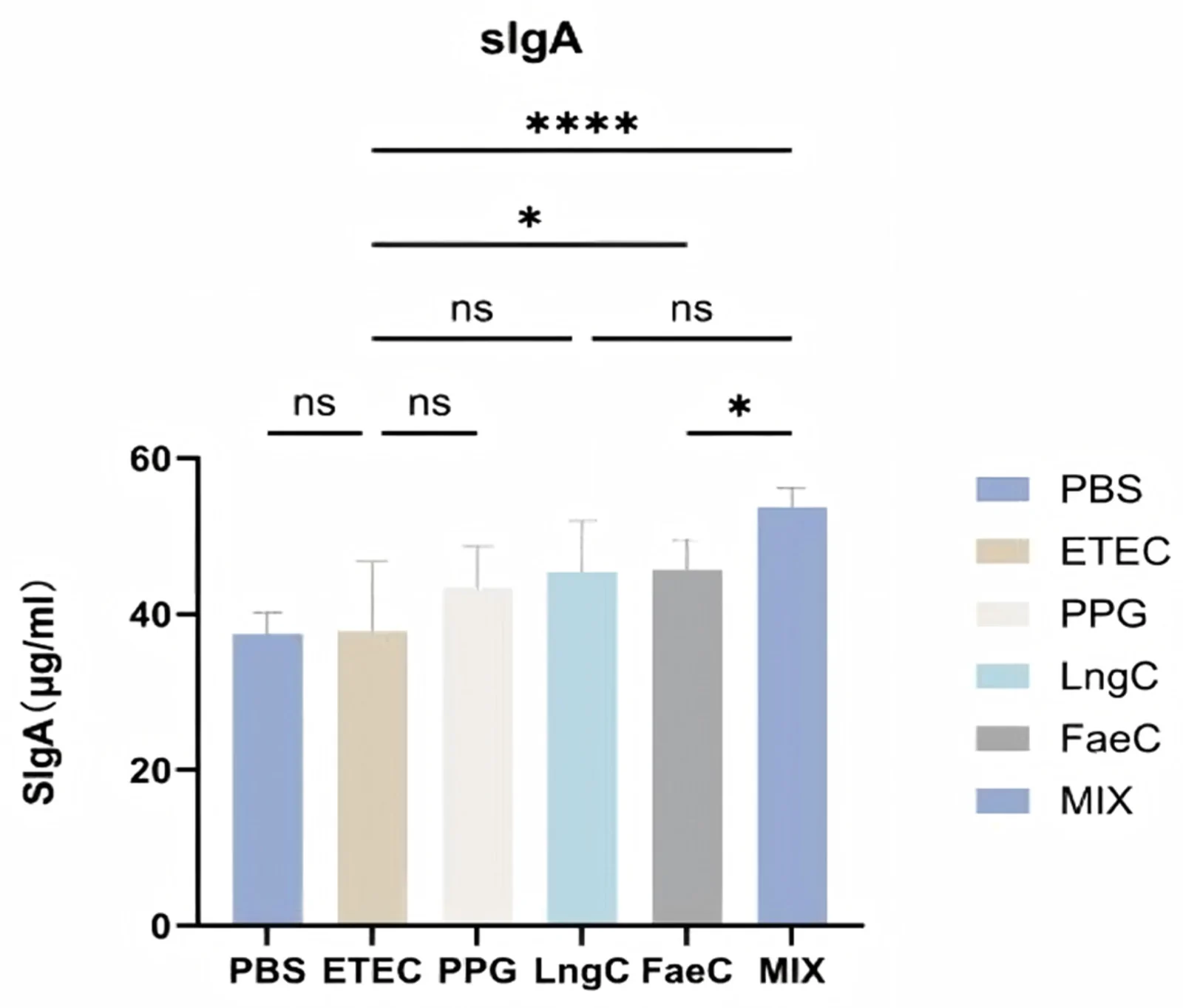
Effect of recombinant *Lactiplantibacillus plantarum* on sIgA levels in the jejunal contents of piglets. Secretory immunoglobulin A (sIgA) concentrations in jejunal contents from each group were measured by ELISA. Data are shown as mean ± SEM (n = 6 piglets per group). Statistical significance was determined by one-way ANOVA compared with the control group; * *p* < 0.05, **** *p* < 0.0001; NS, not significant.

Secretory immunoglobulin A (sIgA) serves as the primary effector molecule of the intestinal mucosal immune barrier. To evaluate the mucosal humoral immune response, the sIgA levels in jejunal contents of piglets from each group were measured by ELISA. As shown in Figure 4, the MIX group exhibited a highly significant increase in sIgA levels compared with the ETEC challenge group (*p* < 0.0001), and the FaeC group also showed a moderate but significant elevation relative to the ETEC group (*p* < 0.05). Furthermore, the sIgA concentration in the MIX group was significantly higher than that in the FaeC group (*p* < 0.05). These results indicate that immunization with the recombinant strains, particularly the combined MIX regimen, effectively stimulated intestinal sIgA secretion, thereby reinforcing the mucosal immune defense against ETEC infection.

The levels of specific IgG antibodies against FaeC and LngC in serum were measured by ELISA and are presented in Supplementary Figure S4. The anti-FaeC IgG levels were significantly higher in the FaeC and Mix groups than in the PBS and ETEC groups (*p* < 0.05; Supplementary Figure S4A). Similarly, the anti-LngC IgG levels were significantly higher in the LngC and Mix groups than in the PBS and ETEC groups (*p* < 0.05; Supplementary Figure S4B).

### 3.4. Recombinant Strains Modulate Serum Cytokine Levels in Piglets

**Figure 5:**
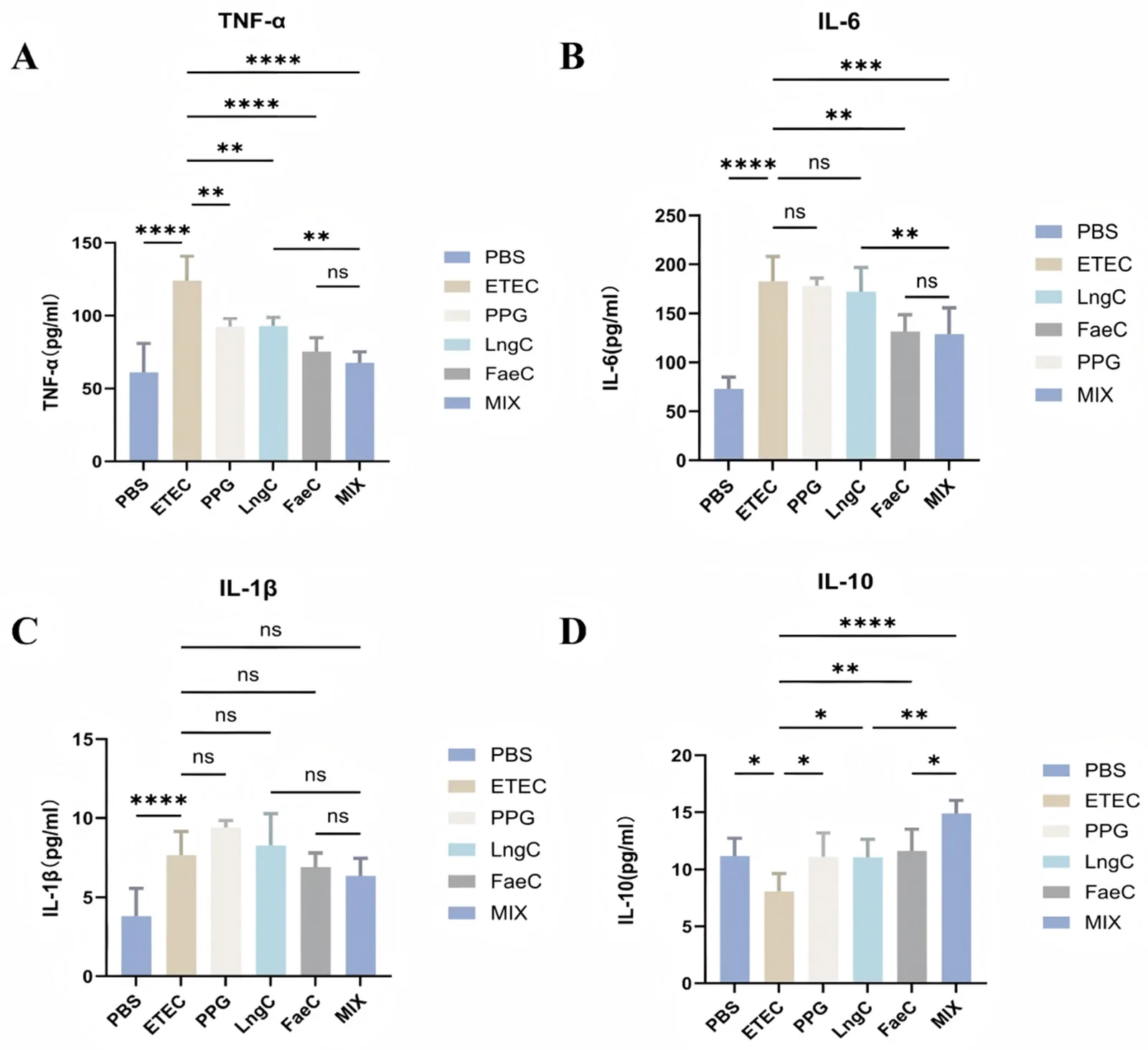
Effects of recombinant *Lactiplantibacillus plantarum* on serum cytokine levels in piglets. Concentrations of TNF-α (**A**), IL-6 (**B**), IL-1β (**C**), and IL-10 (**D**) in piglet serum from each group were determined by ELISA. Data are shown as mean ± SEM (n = 6 piglets per group). Statistical significance was assessed by one-way ANOVA compared with the control group; * *p* < 0.05, ** *p* < 0.01, *** *p* < 0.001, **** *p* < 0.0001; NS, not significant.

To further evaluate the systemic immune response, serum levels of pro-inflammatory cytokines (TNF-α, IL-6, and IL-1β) and the anti-inflammatory cytokine IL-10 were measured by ELISA in each group (Figure 5A–D). As shown in Figure 5A, compared with the ETEC group, serum TNF-α levels were significantly downregulated in the MIX group (*p* < 0.0001), the LngC group (*p* < 0.01), and the FaeC group (*p* < 0.0001). Additionally, the MIX group exhibited significantly lower TNF-α levels than the LngC group (*p* < 0.01). For IL-6 (Figure 5B), the MIX group showed a significant decrease compared with the ETEC group (*p* < 0.001), followed by the FaeC group (*p* < 0.01). The LngC group also exhibited a reduction, although the difference did not reach statistical significance. Regarding IL-1β (Figure 5C), no statistically significant differences were observed among all groups compared with the ETEC group (*p* > 0.05). Notably, as presented in Figure 5D, IL-10 levels were significantly upregulated in the MIX group (*p* < 0.0001), as well as in the FaeC (*p* < 0.01) and LngC (*p* < 0.05) groups, relative to the ETEC group. Furthermore, the MIX group showed significantly higher IL-10 levels than both the LngC and FaeC groups (*p* < 0.01). Collectively, these results demonstrate that immunization with recombinant strains differentially modulates systemic cytokine profiles. The marked differences in inflammatory cytokine levels between the PBS and ETEC groups confirmed the validity of the experimental challenge model. Notably, the combined immunization (MIX group) exerted a more pronounced effect in downregulating pro-inflammatory cytokines (TNF-α and IL-6) while simultaneously upregulating the anti-inflammatory cytokine IL-10, compared with the single-strain immunization regimens.

### 3.5. Recombinant Strains Induce Cellular Immune Responses in Piglets

To evaluate the cellular immune responses elicited by the recombinant strains, flow cytometry was performed to analyze lymphocyte subsets in lamina propria lymphocytes (LPL), mesenteric lymph nodes (MLN), and spleen (SP) from piglets in each group.

#### 3.5.1. Immune Cell Populations in Lamina Propria Lymphocytes (LPL)

**Figure 6:**
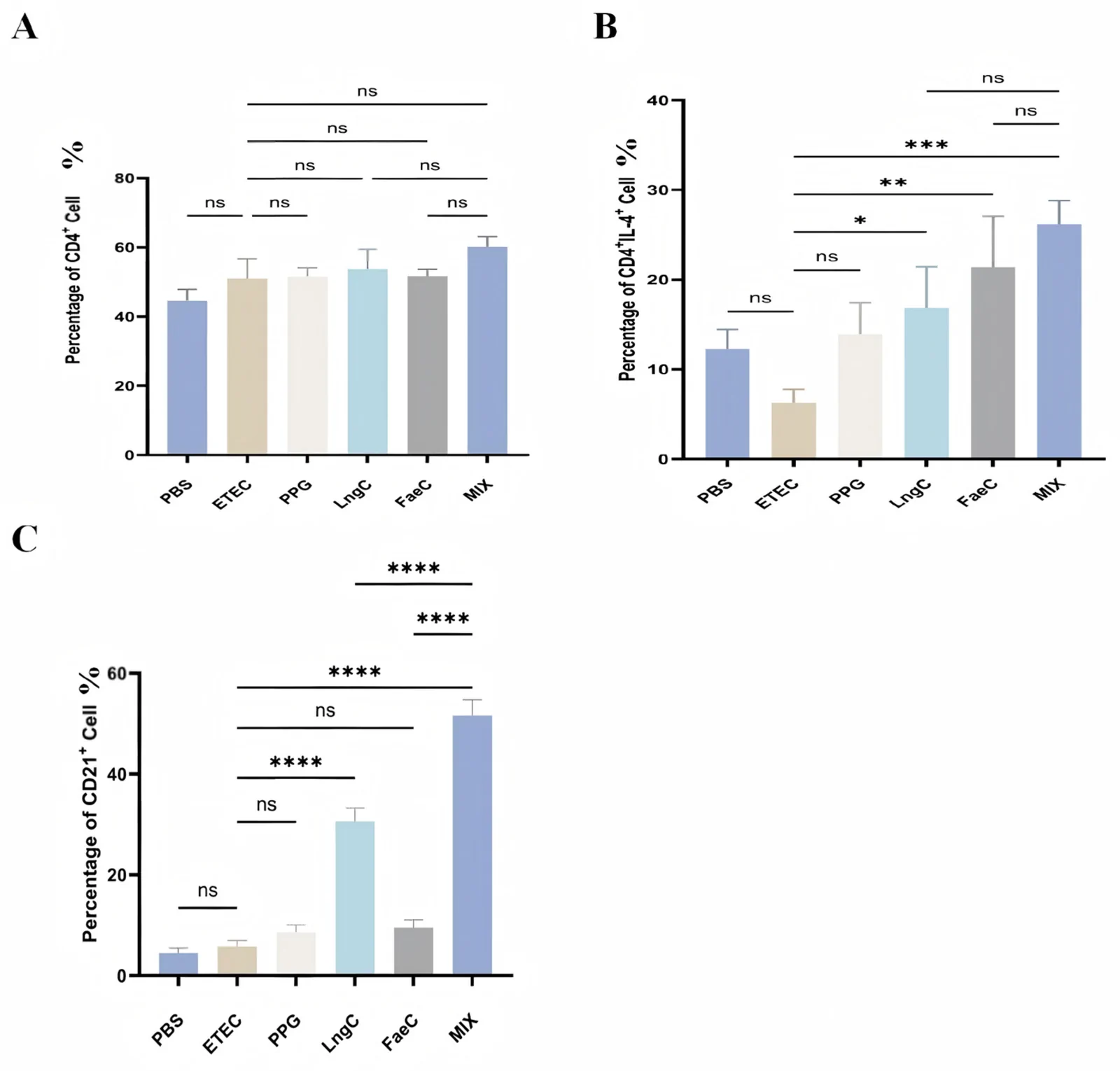
Activation of T cells, B cells, and dendritic cells in piglet lamina propria lymphocytes (LPLs) by recombinant *Lactiplantibacillus plantarum*. LPLs were isolated from each group, and the proportions of CD4^+^T cells (**A**), CD4^+^IL-4^+^T cells (**B**), and CD21^+^B cells (**C**) in LPLs were determined by flow cytometry. Data are shown as mean ± SEM (n = 6 piglets per group). Statistical significance was assessed by one-way ANOVA compared with the control group; * *p* < 0.05, ** *p* < 0.01, *** *p* < 0.001, **** *p* < 0.0001; NS, not significant.

As shown in Figure 6A, no significant differences in the percentage of total CD4^+^ T cells in LPL were observed among all groups compared with the ETEC group (*p* > 0.05), nor between any of the immunized groups (*p* > 0.05), indicating that the overall CD4^+^ T-cell population was not altered by ETEC infection or immunization. For the Th2-type CD4^+^IL-4^+^ T-cell subset (Figure 6B), the MIX group exhibited a highly significant increase compared with the ETEC group (*p* < 0.001), while both the LngC and FaeC groups showed moderate increases (*p* < 0.05). No statistically significant differences were observed among the immunized groups (*p* > 0.05). Regarding CD21^+^ B-cell populations in LPL (Figure 6C), compared with the ETEC group, the LngC and MIX groups displayed significantly increased percentages (*p* < 0.0001). Moreover, the MIX group showed a significantly higher percentage than the LngC group (*p* < 0.0001). Collectively, these results indicate that oral immunization with the recombinant strains effectively increased the proportions of Th2 cells and B cells in the intestinal lamina propria, thereby facilitating processing and immune signal transduction in the gut mucosal immune system.

#### 3.5.2. Immune Cell Populations in Mesenteric Lymph Nodes (MLN)

**Figure 7:**
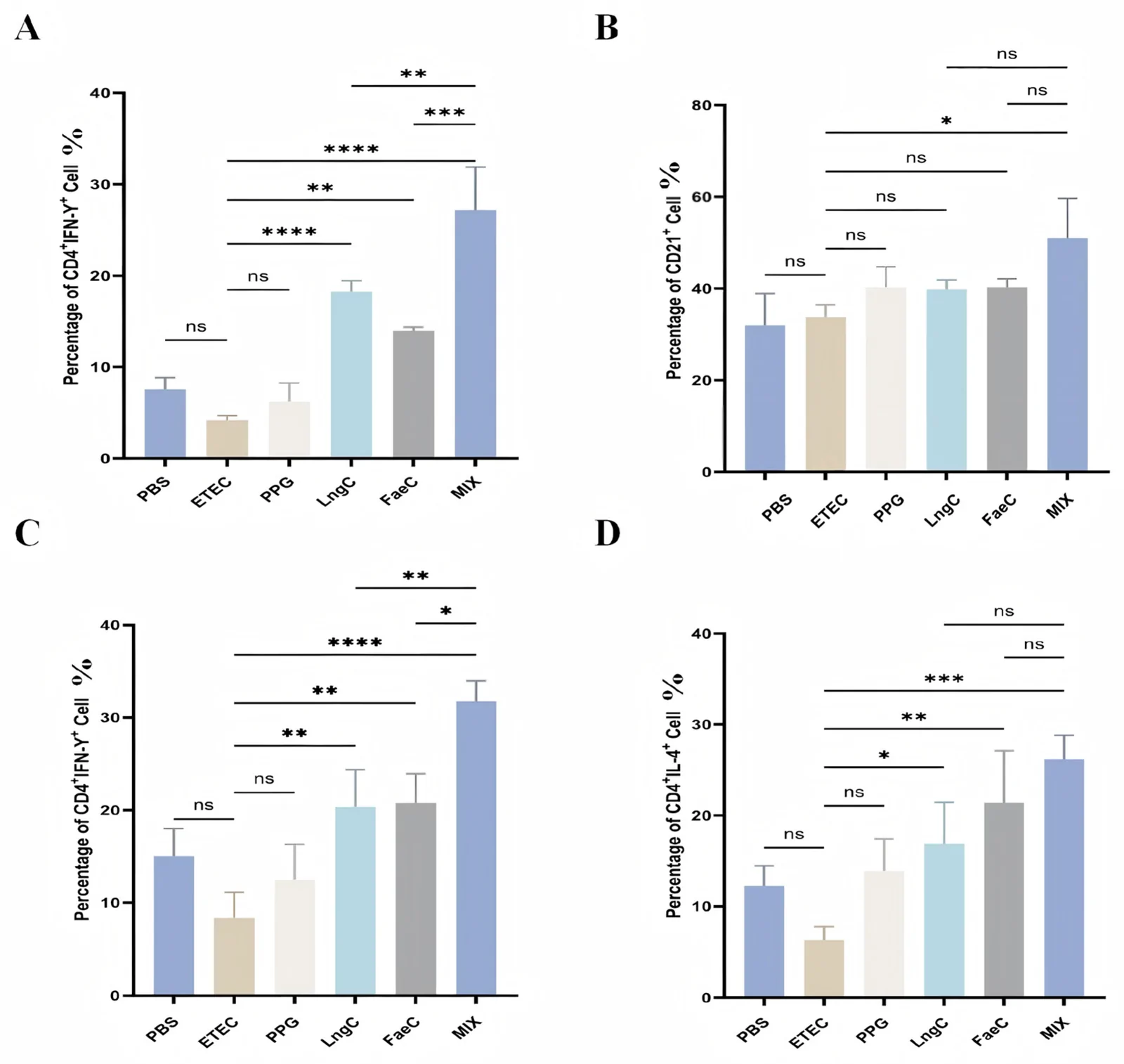
Activation of T and B cells in mesenteric lymph nodes (MLNs) and spleen of piglets by recombinant *Lactiplantibacillus plantarum*. Cells were isolated from the MLNs and spleen of each group. The proportions of CD4^+^IFN-γ^+^T cells (**A**) and CD21^+^B cells (**B**) in MLNs, as well as CD4^+^IFN-γ^+^T cells (**C**) and CD4^+^IL-4^+^T cells (**D**) in the spleen, were determined by flow cytometry. Data are shown as mean ± SEM (n = 6 piglets per group). Statistical significance was assessed by one-way ANOVA compared with the control group; * *p* < 0.05, ** *p* < 0.01, *** *p* < 0.001, **** *p* < 0.0001; NS, not significant.

In MLN (Figure 7A), the percentages of CD4^+^IFN-γ^+^ Th1 cells were significantly elevated in the LngC and MIX groups compared with the ETEC group (*p* < 0.0001) and in the FaeC group (*p* < 0.01). Furthermore, the MIX group exhibited significantly higher Th1 cell percentages than both the LngC group (*p* < 0.05) and the FaeC group (*p* < 0.01). For CD21^+^B cells in MLN (Figure 7B), only the LngC group showed a significant increase compared with the ETEC group (*p* < 0.05); the FaeC and MIX groups showed no statistically significant differences. These findings suggest that the recombinant strains, particularly the combined MIX regimen, effectively induced cellular immune responses in the mesenteric lymph nodes.

#### 3.5.3. Immune Cell Populations in Spleen (SP)

In the spleen (Figure 7C), the MIX group exhibited a highly significant increase in CD4^+^IFN-γ^+^ Th1 cells compared with the ETEC group (*p* < 0.0001), while both the LngC and FaeC groups showed significant increases (*p* < 0.01). Additionally, the MIX group showed significantly higher Th1 cell percentages than both the LngC and FaeC groups (*p* < 0.01). For CD4^+^IL-4^+^ Th2 cells in the spleen (Figure 7D), the MIX group displayed a highly significant increase (*p* < 0.001), and both the LngC and FaeC groups showed significant elevations (*p* < 0.01) compared with the ETEC group. Collectively, these results demonstrate that oral administration of the recombinant strains effectively promoted the differentiation of Th1 and Th2 cells in the spleen, indicating the induction of systemic cellular immune responses.

## 4. Discussion

In this study, two recombinant Lactiplantibacillus plantarum NC8 strains were constructed using the anchoring vector pPG612.1 to surface-display the ETEC F4 fimbrial subunit FaeC and the outer membrane protein LngC, respectively. The results indicate that these recombinant probiotics induced local mucosal and systemic immune responses, attenuated ETEC-induced intestinal injury, and alleviated inflammatory responses. Notably, combined immunization with the two strains was more effective than single-strain immunization in modulating inflammatory responses and enhancing immune responses under the conditions tested, suggesting a potential synergistic effect.

The successful expression of FaeC and LngC on the bacterial surface without compromising growth performance is a critical prerequisite for vaccine development. The stability of the recombinant strains’ growth curves, comparable to that of the wild-type NC8, confirms that the heterologous protein expression and plasmid maintenance impose no significant metabolic burden on the host bacterium. This is consistent with previous reports indicating that *Lactiplantibacillus plantarum NC8*, due to its thin cell wall and absence of native plasmids, serves as a robust platform for surface display of foreign antigens [18]. The ability to anchor antigens on the bacterial surface is particularly advantageous for mucosal vaccination, as it facilitates direct delivery of the immunogen to the gut-associated lymphoid tissue (GALT), mimicking natural ETEC colonization without causing disease.

A key finding of this study is the potent mucosal immune response elicited by the recombinant strains, as evidenced by the significantly elevated secretory immunoglobulin A (sIgA) levels in the jejunal contents of immunized piglets. sIgA is the principal effector molecule of mucosal immunity, forming a protective immune barrier that prevents pathogen adherence and penetration across the intestinal epithelium [19]. The enhanced sIgA production observed in the FaeC and MIX groups is consistent with the well-established role of live vector vaccines in stimulating local humoral immunity [20]. This is particularly relevant for ETEC, given that its pathogenesis begins with colonization of the small intestine. Oral immunization with our recombinant strains likely mimics the natural route of ETEC infection, thereby engaging the intestinal mucosal immune system more effectively than parenteral vaccines, which predominantly induce systemic immunity and are less efficient at generating gut-specific sIgA responses [21].

The immunomodulatory effects of the recombinant strains extend beyond humoral immunity to encompass cellular immune responses. Flow cytometric analysis revealed that immunization significantly promoted the proliferation and differentiation of CD4^+^IFN-γ^+^ (Th1) and CD4^+^IL-4^+^(Th2) T cells in the lamina propria, mesenteric lymph nodes, and spleen. These findings indicate that the recombinant strains successfully activate both cell-mediated and humoral arms of adaptive immunity. Th1 cells, characterized by IFN-γ secretion, play a pivotal role in enhancing the antigen-presenting capacity of macrophages and dendritic cells (DCs) and in activating natural killer (NK) cells, thereby contributing to the elimination of intracellular pathogens and infected epithelial cells [22]. On the other hand, Th2 cells, through IL-4 secretion, are essential for B-cell activation, class switching, and antibody production [23]. The balanced activation of both Th1 and Th2 responses observed in our study suggests that the recombinant strains can orchestrate a comprehensive immune defense against ETEC, which is crucial for both preventing initial colonization and clearing established infections. This dual activation is a notable advantage over vaccines that skew the response toward a single T-helper phenotype.

Another important aspect of our study is the anti-inflammatory effect conferred by the recombinant strains. The ETEC challenge resulted in a significant upregulation of pro-inflammatory cytokines (TNF-α,IL-6, and IL-8) and downregulation of the anti-inflammatory cytokine IL-10 in both intestinal tissues and serum, indicative of a severe inflammatory response. However, immunization with the recombinant strains, particularly in the MIX group, effectively reversed this pattern, reducing the expression of pro-inflammatory mediators while elevating IL-10 levels. Excessive production of pro-inflammatory cytokines is a hallmark of ETEC-induced intestinal inflammation and contributes to tissue damage and diarrhea [24,25,26]. The ability of our recombinant probiotics to dampen this inflammatory cascade is likely mediated through multiple mechanisms. First, by activating mucosal immunity and inducing sIgA, they may reduce ETEC colonization, thereby limiting the source of enterotoxin-induced inflammation. Second, the immunomodulatory properties of *Lactiplantibacillus plantarum NC8* itself, as previously reported [27], may contribute to the regulation of inflammatory responses. Our findings are consistent with those of Wang et al. [28], who demonstrated that *Lactiplantibacillus plantarum* administration reduced serum pro-inflammatory cytokines in ETEC-challenged rabbits, and Zhou et al. [29], who observed similar anti-inflammatory effects in mice. The more pronounced effect of the combined immunization (MIX group) suggests that the simultaneous presentation of two different ETEC antigens (FaeC and LngC) provides broader antigenic coverage and may engage distinct immune pathways, resulting in a more robust regulatory effect on the inflammatory response. This synergistic effect is an important consideration for future vaccine design, advocating for multivalent approaches over monovalent ones.

Histopathological analysis provided direct evidence of the protective efficacy of the recombinant strains. Piglets immunized with NC8/pPG612.1-FaeC and NC8/pPG612.1-LngC exhibited markedly less damage to jejunal villi, with relatively intact mucosal architecture, in contrast to the severe villous atrophy, epithelial necrosis, and inflammatory cell infiltration observed in the ETEC-challenged group. These results are in line with the studies by Rodrigues et al. [30], who reported similar protective effects against ETEC-induced intestinal injury using different intervention strategies.

Despite the promising results, several limitations of this study should be acknowledged. The evaluation of immune remission efficacy was conducted only up to 7 days post-challenge, and the duration of the immune memory response remains to be determined. Long-term studies are needed to assess whether the protection persists beyond the immediate post-challenge period and whether the recombinant strains can effectively establish themselves in the gut microbiota as stable colonizers. Additionally, the optimal immunization dose and regimen, as well as the potential impact of maternal antibodies, which are known to interfere with active immunization in piglets, warrant further investigation.

## 5. Conclusions

Collectively, these findings indicate that the recombinant *Lactiplantibacillus plantarum NC8* strains surface-displaying ETEC FaeC and LngC antigens effectively activated intestinal mucosal immune responses, attenuated intestinal inflammation, and alleviated intestinal injury in piglets. The combined immunization strategy was more effective than single-strain immunization, highlighting the potential of multivalent probiotic-based vaccines. These results provide a scientific basis for the further development of oral probiotic-based vaccines against ETEC infection in swine.

## Figures and Tables

**Table 1:** Primer sequences.

Primer	Restriction Enzyme Cutting Site	Sequence (5′-3′)
FaeC-F	*Nco*I	CCACCATGGTGATGAAAAAAGCATTCTTATT
FaeC-R	*Asc*I	ACCGGCGCGCCCTACTGATAAGTGACAACAA
Lngc-F	*Nco*I	CCACCATGGATGAGAGCAAAATGGGGTGTTT
Lngc-R	*Asc*I	ACCGGCGCGCCCTCATTGAGAGTCGATAACCA

**Table 2:** qPCR primer sequences.

Primer	Sequence (5′-3′)
GapDH	TGGTGAAGGTCGGAGTGAAC
GapDH	GGAAGATGGTGATGGGATTTC
TNF-α-F	CCACGCTCTTCTGCCTACTGC
TNF-α-R	GCTGTCCCTCGGCTTTGAC
IL-6-F	CTGGCAGAAAACAACCTGAACC
IL-6-R	TGATTCTCATCAAGCAGGTCTCC
IL-8-F	TAGGACCAGAGCCAGGAAGA
IL-8-R	AGCAGGAAAACTGCCAAGAA
IL-10-F	CTTCTGATCCAGCCTTCGTG
IL-10-R	CTTCCACAGCTGGAGACTTTAG

## Data Availability

The datasets generated and analyzed during this study are available from the corresponding author upon reasonable request.

## Supplementary Materials

The following supporting information can be downloaded at: https://www.mdpi.com/article/doi/s1, Figure S1. Agarose gel electrophoresis analysis for the construction of recombinant Lactobacillus plantarum strains; Figure S2. Intestinal morphology of piglets in the experimental groups; Figure S3. Diarrhea score and ETEC excretion volume; Figure S4. Specific antibody test.
