# Supplementary material for: Construction of Recombinant Lactiplantibacillus plantarum NC8 Expressing ETEC FaeC and LngC and Preliminary Evaluation of Immune Responses in an ETEC-Challenged Piglet Model
Source: Vet Sci. 2026 Oct 15;13(10):1098. doi: 10.3390/vetsci13101098 (PMC13709613; doi:10.3390/vetsci13101098)
Supplement: Supplementary file 1 [file vetsci-13-01098-s001.zip › vetsci-4576935-supplementary figure 1-4.pdf]

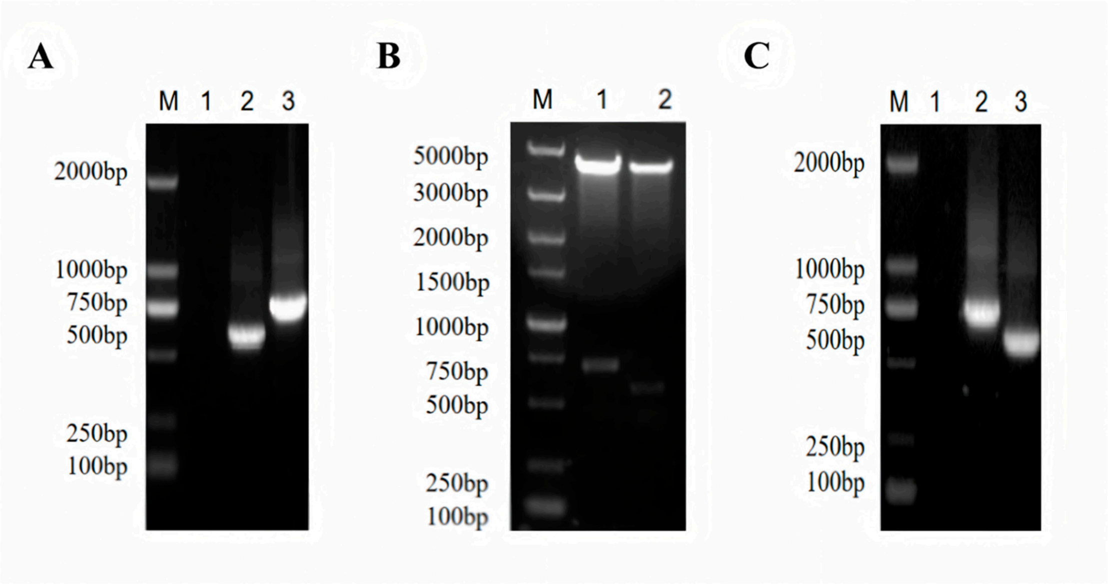

Figure S1. Agarose gel electrophoresis analysis for the construction of recombinant Lactobacillus plantarum strains. (A) PCR amplification of the two target gene fragments (FaeC and LngC). (B) Double restriction enzyme digestion verification of the recombinant plasmids, pPG612.1-FaeC and pPG612.1-LngC. (C) PCR identification of the target genes in the recombinant *Lactiplantibacillus plantarum* NC8 strains.

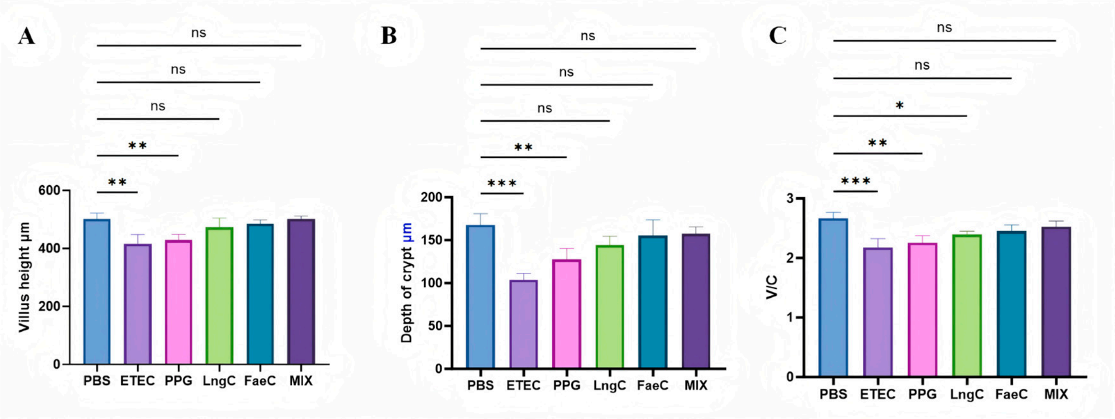

Figure S2. Intestinal morphology of piglets in the experimental groups. (A) Villus height in the intestine of piglets in each group. (B) Crypt depth in the intestine of piglets in each group. (C) Villus height-to-crypt depth ratio (V/C) in the intestine of piglets in each group. Data are presented as mean ± SEM (n = 6 piglets per group). *P < 0.05, **P < 0.01 versus the PBS group. Villus height and crypt depth are expressed in micrometers (μm). The intestinal segments examined were jejunum. Abbreviations: V/C, villus height/crypt depth; SEM, standard error of the mean.

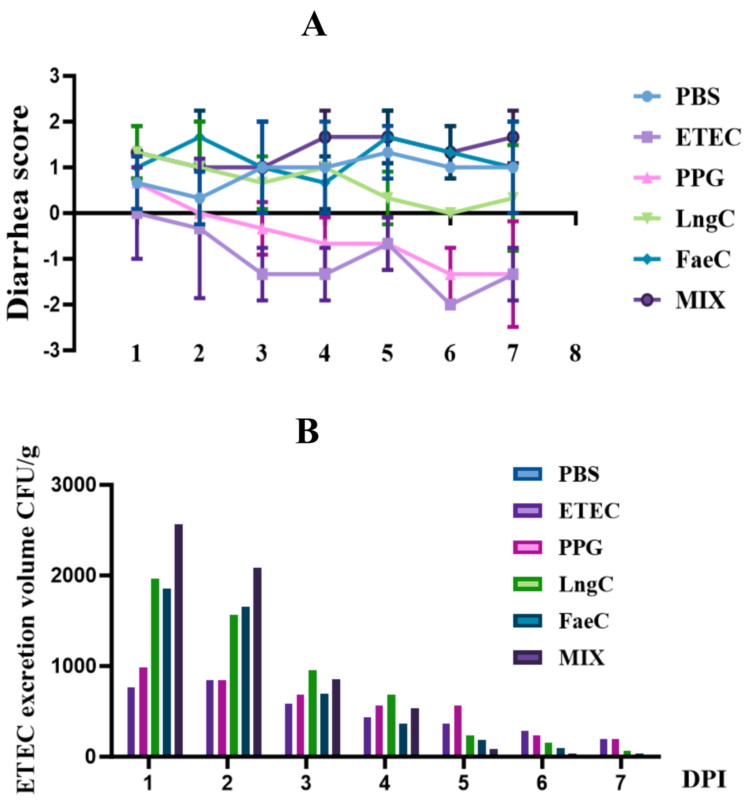

Figure S3. Diarrhea score and ETEC excretion volume. A: Score the appearance of the feces from each group of piglets; B: The excretion situation of ETEC in piglets' feces. Data are presented as mean ± SEM (n = 6 piglets per group).

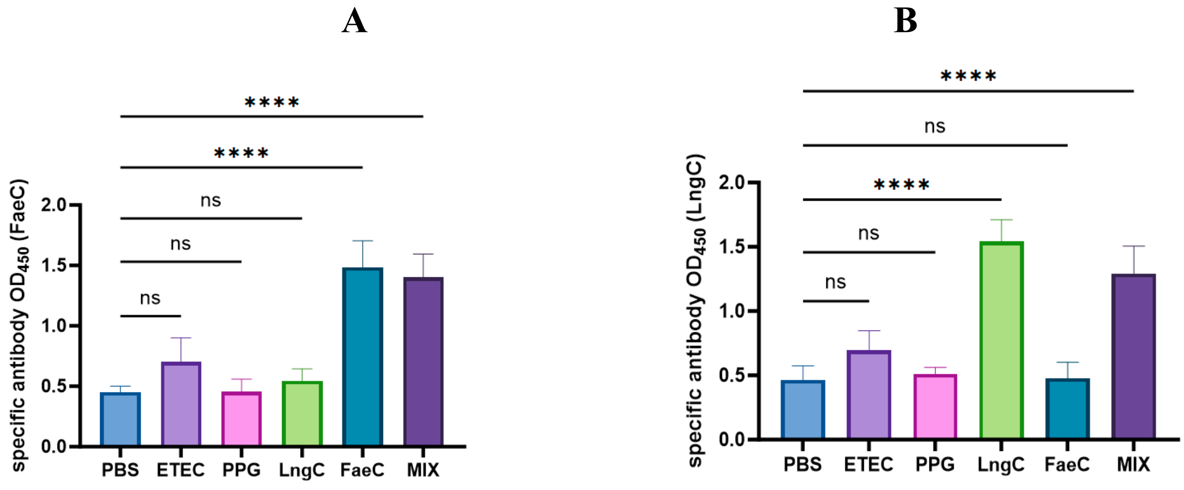

Figure S4. Specific antibody test. A: The detection result of the specific antibody FaeC; B: The test result of the specific antibody LngC. Data are presented as mean ± SEM (n = 6 piglets per group). *P < 0.05, **P < 0.01, ***P < 0.001, ****P < 0.0001; NS, not significant versus the PBS group.
