# Supplementary material for: Construction of Recombinant Lactiplantibacillus plantarum NC8 Expressing ETEC FaeC and LngC and Preliminary Evaluation of Immune Responses in an ETEC-Challenged Piglet Model
Source: Vet Sci. 2026 Oct 15;13(10):1098. doi: 10.3390/vetsci13101098 (PMC13709613; doi:10.3390/vetsci13101098)
Supplement: Supplementary file 1 [file vetsci-13-01098-s001.zip › vetsci-4576935-supplementary figure 5.pdf]

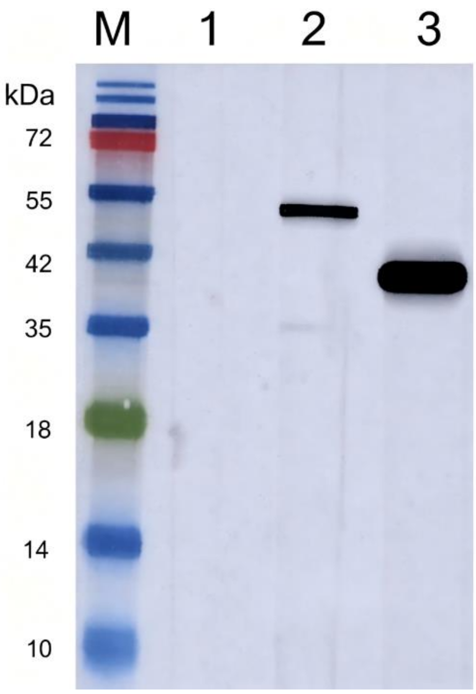

M: protein Marker, 1:NC8 control, 2:NC8/pPG612.1-FaeC,3:NC8/pPG612.1-LngC
